# Acceptability and preferences for dual-active ingredient long-lasting insecticidal nets in rural Tanzania: a mixed-methods study

**DOI:** 10.1186/s12936-025-05723-w

**Published:** 2025-12-15

**Authors:** Eliud Andrea Lukole, Stacie Gobin, Jackie Cook, Jacques Derek Charlwood, Jacklin F. Mosha, Nancy S. Matowo, Elizabeth Mallya, Tatu Aziz, Justina V. Mosha, Jacklin Martin, Mark Rowland, Immo Kleinschmidt, Alphaxard Manjurano, Franklin W. Mosha, Jayne Webster, Natacha Protopopoff

**Affiliations:** 1 Department of Parasitology, National Institute for Medical Research, Mwanza Medical Research Centre, Mwanza, Tanzania; 2 Department of Disease Control, London School of Hygiene and Tropical Medicine, London, UK; 3 Gobin Global, LLC, Asheville, NC USA; 4 International Statistics and Epidemiology Group, Department of Infectious Disease Epidemiology and International Health, London School of Hygiene and Tropical Medicine, London, UK; 5 Global Health and Tropical Medicine, Instituto de Hygiene E Medicinal Tropical, Universidade NOVA de Lisboa, Lisbon, Portugal; 6 Kilimanjaro Christian Medical University College, Moshi, Tanzania; 7 Wits Research Institute for Malaria, School of Pathology, Faculty of Health Sciences, University of the Witwatersrand, Johannesburg, South Africa; 8 Southern African Development Community Malaria Elimination Eight Secretariat, Windhoek, Namibia; 9 Swiss Tropical and Public Health Institute, Basel, Switzerland

## Abstract

**Background:**

The World Health Organization (WHO) recommends dual-active ingredient long-lasting insecticidal nets (dual-AI LLINs) for protection against malaria in areas with insecticide resistance. The effectiveness of LLINs, however, depends on user compliance, influenced by community perceptions of malaria, prevention methods, and the acceptability of LLINs. Understanding these factors is essential for the success of large-scale implementation.

**Methods:**

This study was part of the two cluster-randomized controlled trials (RCTs) evaluating the efficacy of dual-AI LLINs on malaria indicators in Muleba and Misungwi districts, Tanzania. Polyethylene and polyester rectangular LLINs were distributed in Muleba (Olyset Plus and Olyset Net) in 2015 and in Misungwi (Olyset Plus, Royal Guard, Interceptor G2, and Interceptor) in 2019. A mixed-methods approach was used to assess users’ acceptability, preferences, and perceptions, and identifying barriers to consistent use. Quantitative data were collected from 14,475 households, while qualitative data came from 36 focus group discussions and 44 in-depth interviews. A thematic analysis was applied using a deductive approach, guided by the study’s conceptual framework. Descriptive statistics were used for quantitative analysis.

**Results:**

LLIN usage and acceptability were influenced by their availability in the households, the physical integrity, side-effects, nuisance from mosquito bites and perceived malaria risk. Olyset Plus was slightly favoured over Olyset net in Muleba due to the perception that the insecticide had a stronger effect (72% vs 63%), p = 0.1395. In Misungwi, net acceptability, measured by the proportion reporting LLINs as no longer protective, varied significantly by net type at 24, 30, and 36 months (p < 0.0001). In Misungwi, Olyset Plus had the highest overall dissatisfaction (15.1%), followed by Royal Guard (12.0%) while Interceptor G2 and Interceptor had the lowest (7.0–7.6%). In Misungwi, 86% (2338/2736) preferred polyester nets over polyethylene due to better comfort and durability. Adverse effects (itching, skin irritation) were reported more frequently for Royal Guard and Interceptor (48–55%). Bed bug infestations were found in 19–29% of study nets, averaging 15 bugs/net, negatively influenced consistent use. LLIN repurposing was more common in Misungwi (35%) than Muleba (19%). Preferences skewed heavily toward blue (61% in Muleba vs 93% in Misungwi), rectangular LLINs (61% in Muleba vs 91% in Misungwi) in both sites.

**Conclusion:**

Acceptability and sustained use of dual-AI LLINs are shaped by perceived efficacy, comfort, and net integrity, while barriers like bed bugs and skin irritation reduce use. Addressing non-target pest issues, targeting different groups of users with tailored education, and integrating user perception into LLIN procurement can enhance uptake and impact. It is recommended that manufacturers and policymakers consider these community-informed insights to guide the development and deployment of more acceptable LLINs.

**Supplementary Information:**

The online version contains supplementary material available at 10.1186/s12936-025-05723-w.

## Background

Malaria caused approximately 597,000deaths globally in 2024 [1]. Tanzania is among the four countries with the highest malaria deaths accounting for 4.4% of the global deaths in 2024 [1]. Malaria is highest in the Lake Victoria zone, with the districts of Muleba and Misungwi having an annual incidence rate of 130.9, and 24.3 cases per 1000 population in 2023, respectively [2, 3]. Malaria has a profound economic and social impact, contributing to human suffering and limiting development [4, 5]. Countries with high malaria transmission have been shown to have income levels about one-third lower than those without malaria. Indeed, countries that have eliminated malaria have typically experienced substantially higher economic growth in the following 5 years [6]. LLINs, primarily treated with pyrethroids, have been the cornerstone of malaria prevention for decades [7, 8]. Acceptability of pyrethroid-only long-lasting insecticidal nets (LLIN) has generally been high over the last few decades, with studies suggesting this is due to their perceived effectiveness [9–11]. However, growing resistance to pyrethroid insecticides has reduced the effectiveness of these nets, which may, in turn, impact their acceptability.

In 2017, the World Health Organization (WHO) recommended the deployment of novel LLINs combining a pyrethroid with a synergist piperonyl butoxide (PBO) [12]. In 2023, the WHO further endorsed LLINs with dual active ingredient treated with pyrethroid, with either pyriproxyfen or chlorfenapyr [13] to control malaria-transmitted by insecticide-resistant vectors. In that same year, 360 million pyrethroid-piperonyl butoxide (PBO) LLINs, and 74 million dual active ingredient LLINs were distributed across sub-Saharan Africa (SSA), including Tanzania [1, 14], with the number increasing to 82 million in the second quarter of 2025. Between 2018 and 2025, the proportion of dual-active ingredient long-lasting insecticidal nets (dual-AI LLINs) including PBO LLINs distributed in SSA increased from 3 to 84% of all LLINs distributed [14]. Following the WHO recommendations, the Tanzanian National Malaria Control Programme (NMCP), with the support of the President’s Malaria Initiative (PMI), and Global Fund (GF), has distributed over 2,945,181 PBO LLINs by 2024 [15, 16]. NMCP is also considering the deployment of dual-AI LLIN, such as Interceptor G2, for future LLIN campaigns [16].

Previous studies in Tanzania have examined LLIN user’s attitude [17–20]. Nuisance from mosquito bites was identified as the main factor influencing consistent use. Individuals who used LLINs primarily for malaria protection were more likely to adhere than those who used them mainly to prevent mosquito-biting nuisance especially when mosquito density was low. Hot weather was also to be a major deterrent to LLIN use [21]. While mosquito net manufacturers aim to improve ventilation by enlarging the mesh size, they are constrained by the need to keep the openings small enough to block mosquitoes [22]. Various characteristics of LLINs brands such as durability, texture, mesh size, net size, shape, colour, and insecticidal effectiveness have been found to influence acceptability and usage [9]. The net material also influences user preference and durability. Studies in Kenya [23], India, and Nepal [24] found users preference leaning towards polyester have a softer texture. In contrast polyethylene have a stiffer texture, shrink after washing (making them difficult to tuck under bedding), and have larger mesh sizes which are perceived as allowing mosquito entry. Conversely, a study in Uganda [25] found a slight preference for polyethylene LLINs over polyester. These findings highlight the importance of determining community preferences before procuring LLINs.

A key challenge for malaria control in SSA is not only achieving sufficient LLIN coverage but also addressing the behavioural factors that affect their use. Providing LLINs that are acceptable to users can improve adherence and, ultimately, contribute to the success of malaria control programmes. To address this operational priority, two studies, embedded within cluster–cluster-randomized controlled trials (RCTs), were conducted to assess acceptability and preferences for five types of LLINs with different textiles and insecticidal properties and, to identify barriers to appropriate use.

## Methods

### Study design

**Fig. 1:**
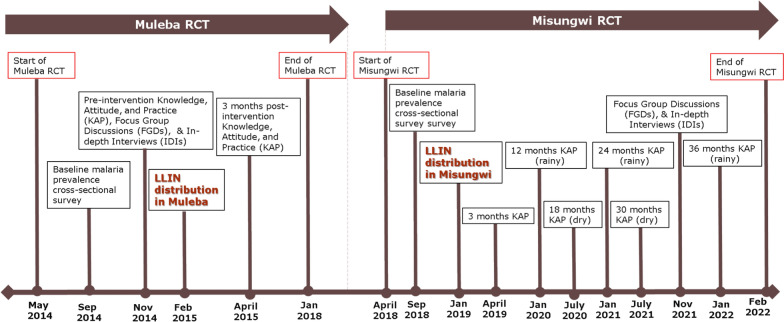
KAP, FGDs, and IDIs data collection timeline

This mixed-method study was part of a two large cluster-randomized controlled trials (RCTs) (Table 1) conducted in Muleba and Misungwi districts. The RCTs aimed to assess the epidemiological and entomological efficacy of different types of dual-AI LLINs against malaria transmitted by resistant mosquitoes [26–28]. In Muleba, the quantitative component of the study included knowledge, attitudes, and practices (KAP) surveys pre- and post-intervention, and a qualitative study pre-intervention (Fig. 1). In Misungwi, KAP questions were included in a survey 3 months post-intervention and in malaria prevalence surveys at 12, 18, 24, 30, and 36 months post-intervention; qualitative data was collected 34 months post-intervention only (Fig. 1).

**Table 1:** RCTs in Muleba and Misungwi districts

RCTs	Muleba RCT: March 2014–December 2017	Misungwi RCT: April 2018–March 2022
Study design	A four-arm, factorial design, cluster-randomized trial (CRT) with 48 clusters (12 clusters per arm) and village/hamlet as the unit of randomization. Each cluster was comprised of 1–6 hamlets (sub-village) with up to 450 houses per cluster The four arms/interventions were: 1. Olyset Plus (permethrin + piperonyl butoxide (PBO) LLIN) 2. Olyset Plus + Indoor Residual Spraying (IRS) with pirimiphos-methyl applied at a dosage of 1–2 g AI/m^2^, provides a residual effect lasting 6–9 months 3. Olyset net + IRS 4. Olyset net [control/reference arm]: (permethrin only LLIN)	A four-arm, superiority design, single-blinded, cluster-randomized trial with 84 clusters (21 clusters per arm) with village/hamlet as the unit of randomization. Each cluster was comprised of 1–8 hamlets (sub-village) of between 150–450 houses in the core area of a cluster The four arms/interventions were: 1. Interceptor G2 (alpha-cypermethrin + chlorfenapyr) 2. Royal Guard (alpha-cypermethrin + pyriproxyfen) 3. Olyset Plus (permethrin + piperonyl-butoxide (PBO)) 4. Interceptor [control/reference arm]: (alpha-cypermethrin)
LLIN distribution	LLINs were distributed from 6th-8th February 2015, and Indoor residual spraying (IRS) took place once in February/March 2015. Approximately 90,000 LLINs were distributed in 30,000 households, with each arm receiving only one type of net	LLINs were distributed from 26th-28th January 2019. A total of 147,230 LLINs were distributed in 42,394 households, with each arm receiving only one type of net
LLIN fabric, colour, shape, and size of distributed LLINs	Polyethylene material, blue in colour, and rectangular in shape (length 180 cm, Height 180 cm, and Width 160 cm)	Polyethylene (Olyset Plus and Royal Guard), polyester (Interceptor G2 and Interceptor), all blue, and rectangular (length 180 cm, Height 180 cm, and Width 160 cm)

### Study sites, and setting

Muleba is the largest district of the Kagera region, located in the northwest of Tanzania on the western shore of Lake Victoria. The population in Muleba is predominantly *Wahaya* (Haya-speaking), who depend on bananas as their staple food and coffee as a cash crop. The district covers an area of approximately 3500 km^2^ at an altitude ranging from 1100 m to 1600 m above sea level, with a population of 637,659 people (2022 population census) [29]. Misungwi is one of the eight districts of the Mwanza region, located on the southern border of Lake Victoria, north-western Tanzania. The population in Misungwi is predominantly *Wasukuma* (Sukuma-speaking), who mainly depend on maize, rice, and cassava as staple food and cotton as a cash crop. Misungwi has a population of 467,867 people, with 48% under 15 years of age. The study areas are described in detail elsewhere [26–28]. Both districts experience year-round malaria transmission, which peak in June/July and December/January. Rainfall occurs from September to December (short rainy season) and from March to May (long rainy season). Previous LLIN universal coverage campaigns in the study sites were conducted in 2011 in Muleba and in 2015 in Misungwi, during which Olyset Nets were distributed. Other LLIN brands present in these districts were provided through antenatal clinics and the School Net Programme (SNP). At baseline (before the study LLINs were distributed), malaria prevalence in the Muleba study area was 65% and 28% of the population reported using any LLIN the night before the survey. In Misungwi, malaria prevalence was 44% and 61% of the population reported using an LLIN [26, 27] (Table 2). Over the course of the trials, study LLIN use and malaria prevalence fluctuated in both settings (Table 2).

**Table 2:** Usage of nets and malaria prevalence over the study period

District/year	Baseline		Year 1-post intervention		Year 2-post intervention		Year 3-post intervention	
	Malaria infection	All LLIN use	Malaria infection	Study LLIN use	Malaria infection	Study LLIN use	Malaria infection	Study LLIN use
Muleba	65.0%	27.5%	39.8%	77.3%	44.3%	56.0%	64.7%	39.0%
Misungwi	44.2%	61%	21.9%	61%	42.1	40.9%	38.5%	22.3%

### Data collection

The selection of respondents for quantitative and qualitative interviews and FGDs included a mix of random and purposive sampling.

### Knowledge, attitudes and practices (KAP)

In Muleba, both baseline and post-intervention KAP surveys were conducted in 16 clusters (4 clusters per study arm) purposively selected based on LLIN usage [high (> 40%) or low (≤ 40%)] and malaria prevalence [high (> 30%) or low (≤ 30%)] (Additional file 1).

At baseline 40 households with children under 15 were selected per cluster. Data were collected on household demographics, LLIN ownership and usage, malaria knowledge, sources of malaria information, personal agency, perceived norms, perceptions of failed LLINs, and treatment-seeking behaviour. Socio-economic variables were not collected at baseline but were captured during the post-intervention survey. For the post-intervention survey, conducted three months after distribution, 48 households were randomly selected per cluster, including households without children under 15. This survey captured broader experiences with Behaviour Change Communication (BCC)/Information Education Communication (IEC) messages and preventive campaigns. Additional questions addressed exposure to social behaviour change communication (SBCC) materials and household involvement in LLIN and IRS campaigns. Pendragon Forms (Pendragon Corporation Software, Libertyville, USA) on Personal Digital Assistants (PDAs) was used to collect data (Table 3).

**Table 3:** Definition of terms

Term	Definition
Adherence to LLINs use	The extent to which individuals or households follow the recommended practices for using LLINs. It specifically involves: consistent use (using the LLIN even during non-peak malaria seasons), proper installation, care and repair and avoiding misuse
Consistent LLIN use	Regular and sustained use of LLINs by individuals or households for protection against malaria mosquitoes. Specifically, it means that people sleep under the net every night. High adherence ensures consistent use, but consistent use alone does not guarantee adherence if the net is not properly maintained or used effectively
Acceptability	The degree to which a LLINs is considered suitable, desirable, and appropriate by the target population, and whether individuals and communities are willing to use and sustain the proper use of nets

In Misungwi, in each of the 84 clusters, at each survey timepoint (3, 12, 18, 24, 30, and 36 post-intervention), 45 households were randomly selected from a census list, except at 3 months where only 10 households were selected per cluster. KAP questions covered similar topics as in Muleba but were shorter and focused on acceptability of nets, perception on nets, favourable features of nets. In addition, in a subset of LLINs presence of bed bug on net as well as numbers were recorded. Surveys were conducted using Android Tablets with Open Data Kit (ODK) software.

In both settings, respondents were primarily household heads, their spouses, or other adults over 18 years of age. Data collection was conducted by field workers who had completed secondary education and received one week of training covering study design, field procedures, and questionnaires.

### In-depth interviews (IDIs) and focus group discussions (FGD)

In each site, participants for IDI and FGD were purposively sampled based on study arm, LLIN usage (high/low), malaria prevalence (high/low), age and sex from baseline survey. In Muleba, participants were selected from 16 clusters (the same clusters involved in-KAP surveys) and in Misungwi from 20 clusters (5 clusters per arm) (Additional file 1).

In both sites, IDIs were conducted with selected district, ward, and village leaders, health facility administrators, Environmental Health Officers (EHOs), community health workers (CHWs) and heads or spouses of households. These interviews explored perceptions of malaria control and prevention interventions, their expected and unexpected effects, and contextual factors affecting the uptake of these interventions. Key informants were contacted directly and invited to choose a convenient time for their interview. The interviews were conducted in locations that ensured privacy and confidentiality for the participants.

FGDs were conducted separately within each demographic group. These groups included married men (26 + years), married women (26 + years), young men (16–25 years), and young women (16–25 years). In Misungwi, an additional group of primary school children (9–15 years) was included. The discussions focused on topics such as knowledge, attitudes, and practices related to malaria prevention and treatment, drivers and barriers to LLIN use, misconceptions about malaria and LLINs, and the acceptability and preferences for LLINs (Table 3).

### General procedures for FGDs and IDIs

The FGDs and IDIs were conducted by two teams of researchers in Muleba in November 2014 and three teams in Misungwi in November 2021. All team members were university graduates fluent in local languages. Each team included one experienced researcher and one or two note-takers. Interviews were led by the experienced researchers, while the note-takers, who received two weeks of intensive training, documented and summarized the interviews. This training covered the study design, ethical treatment of participants, use of semi-structured guides, operation of tape recorders, and note-taking techniques. A semi-structured guide was used to initiate the discussions, covering topics such as malaria knowledge, the purpose of using LLINs, rumours or misconceptions about LLINs, factors influencing their use, non-use, and repurposing, as well as participants’ perceptions of LLIN characteristics and other preventive practices. Participants were encouraged to speak freely around the topics and to express their thoughts and feelings. The lead researcher (EL) observations and pictures and geo-locations were also included to authenticate the findings from the IDI and FGDs.

In Misungwi, due to the use of four distinct LLIN brands, participants were shown each brand during discussions to help them accurately identify the one they received and provide informed feedback. The qualitative feedback on the dual-AI LLINs is particularly relevant in the Misungwi study, as data was collected post-intervention. All FGDs and IDIs in both sites were conducted in *Kiswahili, Kihaya,* and/or *Kisukuma*.

### Data management and analysis

In Muleba, quantitative data were transferred from PDAs to a Microsoft Access database. In Misungwi, data were uploaded from tablets to a server and downloaded as comma-delimited files. All data were then imported into STATA version 15 (Stata Corporation, College Station, Texas, USA). Data from all survey points were combined for analysis at each study site. Quantitative data analysis involved using descriptive statistics means, and proportions.

Interviews and discussions were primarily conducted in the mornings, lasting between 30 and 60 min. Each afternoon, a detailed summary of the main points by theme and study arm was created using the notes taken. In cases of discrepancies, digital recordings were reviewed. In Muleba, recordings of FGDs and IDIs were transcribed *verbatim* from Kihaya/Kiswahili and then translated into English. In Misungwi, recordings were directly transcribed and translated into English by an experienced translator. A sample of IDIs and FGDs was double-checked by the lead researcher (EL) before analysis.

Data were imported into NVivo version 14.0. For coding, text searching, and data merging. Triangulation of qualitative and quantitative data was also used to validate and interpret emerging patterns. A thematic analysis was applied using a deductive approach, guided by the study’s conceptual framework. Themes and sub-themes were examined considering the frequency, intensity, and diversity of views expressed. This flexible approach allowed for the identification, analysis, and reporting of patterns within the data. Matrices were constructed for each main theme, incorporating relevant data from IDIs and FGDs. These matrices facilitated cross-comparisons across interviews and groups to assess the strength of acceptability. All data were analysed using this method. Relevant quotes were selected to support and illustrate the findings.

To maintain anonymity, FGD participants were assigned random numbers, and IDI participants were coded. Quotes were reported using participant numbers or IDI codes, along with the district and cluster category, whether from areas of low or high malaria prevalence or low or high LLIN use.

### Ethics

#### Muleba trial

The trial was approved by the ethics review committees of the Kilimanjaro Christian Medical University College, the London School of Hygiene & Tropical Medicine, and the Tanzanian Medical Research Coordinating Committee (NIMR/HQ/R.8a/VolIX/1803). A trial steering committee reviewed progress. Written informed consent was provided by adult participants.

#### Misungwi trial

Ethical approval for the RCT was secured from the institutional review boards of the Tanzanian National Institute for Medical Research (NIMR/HQ/R.8a/Vol.IX/2743), Kilimanjaro Christian Medical University College (2267), London School of Hygiene & Tropical Medicine (14,952; 14,952-1), and the University of Ottawa (H-05–19-4411). Written informed consent was obtained from adult participants. For children, written assent forms were completed by both the children and their parents or guardians prior to the discussions.

## Results

A total of 1,397 households were visited during the KAP surveys in Muleba, with 88% (560/640) households interviewed (consented) pre-intervention and 78% (593/768) households consented post-intervention. In Misungwi, the KAP questions were embedded in prevalence surveys and included responses from 13,322 households across six post-intervention surveys. Qualitative data were collected through 44 IDIs (14 conducted in Muleba and 30 in Misungwi), and 36 FGDs, with 17 in Muleba and 19 in Misungwi, as detailed in Table 4.

**Table 4:** FGDs, IDIs, and KAP done in Muleba, and Misungwi

Study site	Muleba		Misungwi	
In-depth interviews (IDIs) groups	Number of IDIs conducted	Number of people interviewed in IDIs	Number of IDIs conducted	Number of people interviewed in IDIs
District health workers	2	2	1	1
Environmental health officers (EHO)	N/A	N/A	4	4
In-charges of dispensaries	N/A	N/A	5	5
Villages and ward leaders	8	8	5	5
Community health workers (CHW)	4	4	6	6
Household heads and spouses	N/A	N/A	9	9
Focus group discussions (FGDs) groups	Number of FGDs conducted	Number of people in discussion	Number of FGDs conducted	Number of people in discussion
Married men (26 + years)	5	41	3	34
Married women (26 + years)	5	45	4	46
Young men (16–25 years)	4	30	4	40
Young women (16–25 years)	3	27	2	24
Primary school boys (9–15 years)	N/A	N/A	2	24
Primary school girls (9–15 years)	N/A	N/A	4	48

### Knowledge, and awareness about malaria

In both districts, the majority of respondents correctly identified mosquito bites as the cause of malaria (92.5% in Muleba, 86.6% in Misungwi) (Additional file 2). However, a small percentage of respondents still hold misconceptions such as attributing malaria to superstitions (11.4%) in Misungwi. This misconception was significantly higher in households where the head of the household (p = 0.0001) or the wife (p = 0.0051) had not attained formal education. No significant differences in misconception were observed by socio-economic status (p = 0.1115).

In Muleba, sun exposure was thought to be associated with malaria, with 7.1% of respondents holding this belief at baseline, increasing to 16.9% post-intervention. This perception remained consistent across sex (p = 0.5097), age group (p = 0.4768), socio-economic status (p = 0.5207), and education level (p = 0.5464). Most respondents were aware that using LLINs helps prevent malaria, with (91% in Muleba) recognizing their importance (Additional file 2). However, the practical application of this knowledge varied, particularly during hot seasons (Box 1).

Box 1: Malaria knowledge, and awareness (quotes)

**Table:** 

KII 1: Muleba, district level Respondent (R): Many people do not use LLINs during hot seasons, and it is that time when mosquitoes and malaria are peaking…Sensitization messages need to be intensified for a change of behaviour to use LLINs all the time
FGD 1: Muleba, high malaria cluster P5: Malaria is caused by mosquitoes. Not all mosquitoes cause malaria, the most known to cause malaria are the female anopheles…Mosquito bites all the time, but the ones that transmit malaria bite late at night
IDI 1: Misungwi, high malaria prevalence cluster R: I own a restaurant (many people gather here), I overhear them saying that my child is suffering from malaria, so malaria is high in our community, and the children suffer most R: We usually use nets during the heavy rain season, but we were advised to use them year-round since mosquitoes are still present. Some people, when summer begins, take down their mosquito nets and store them away R: I have learned that using mosquito nets helps you a lot not to catch malaria, and if you don’t use it you will suffer from malaria since there are a lot of mosquitoes in here….it also can protect you from snakes, and other dangerous insects like spiders
FGD 2: Misungwi, high malaria and high net use cluster P6: The first protection we take is using the best quality mosquito net not like the mosquito nets that we received before which had big holes. Also, the family must drink clean water Facilitator: How is malaria transmitted from one person to the other? P7: Malaria spread through the air from one person to the other. Yes, when one sneezes while he/she has malaria fever, when that air reaches you it means you will also catch malaria, yes it spreads through the air
FGD 3: Misungwi, high net use cluster P2: There is one family in our neighborhood whose child had malaria, and they delayed taking him to the hospital after three days of taking antibiotics he did not recover so they took him to *Mitindo* Hospital [district hospital] where tests revealed he had chronic malaria. Unfortunately, despite the hospital’s efforts, the child passed away shortly after. So, we can say it is a big problem that’s the reason we are losing many children because of malaria P7: It’s not possible to recognize that I have malaria, but I might have symptoms like fever, headache, joint pains, chills, and tiredness. Another symptom that I never understand is stomach ache because once I go for the test it is confirmed that I have malaria
FGD 4: Misungwi, low malaria and low net use cluster P5: What I know is if the mosquito bites a breastfeeding woman if that woman gets malaria, she will be able to infect the child

P = participant in FGD; R = Respondent in IDI

In the qualitative studies (Box 1) despite high awareness about malaria, some respondents, especially in Misungwi, believed that malaria could be spread through the air or from a breastfeeding mother to her child. There was skepticism about whether malaria could be a direct cause of death, with some attributing deaths related to malaria to other causes, including witchcraft.

“…*At funerals, if the cause of death is stated as malaria, there is often a collective murmur of disbelief, with people questioning, "Has only malaria killed him/her?" They struggle to accept that malaria alone could be the cause of death”. (P9: FGD Misungwi, low malaria, and low net use cluster)*

### Determinants of LLIN use

During the baseline survey in Muleba, net usage among respondents significantly varied by sex, with more women reporting using nets than men (p = 0.0016), but not by age group (p = 0.2955) nor education status (p = 0.6027). The main reasons given for not using nets at baseline were not having enough nets (56.5%) or available nets being worn out/too torn (29.4%) (Additional file 2). Three months after the net distribution, 94% of people reported using nets (Additional file 2), and this usage did not differ by socio-economic status (p = 0.1966), or sex (p = 0.9990) or education level (p = 0.8045). In the qualitative study, the main determinant of consistent LLIN use included having sufficient LLINs within the household, the perception that LLINs decrease malaria risk, avoiding mosquito bites, and protection from other pests like spiders, snakes, and rodents.

*“….Many households lack enough nets, and the ones they have are badly torn. You know, in the last campaign, only two bed nets were provided per household, regardless of the number of sleeping places or people”. (IDI 2, Muleba, low malaria & low net use cluster).*

*“They are using nets to prevent mosquitoes; it has become their habit nowadays. Also, the level of disease has decreased because mosquito nets protect them. Even in my family, there was frequent fever but once we started using mosquito nets fever has decreased” (IDI 3: Misungwi, high malaria cluster)*

*“One day, my wife and I visited a relative in a distant village. At night, they set up a net where we were allocated to sleep. To our surprise, when we woke up in the morning, we saw a tiny snake on the roof of the net. Since then, I always use a net when I sleep, whether there are mosquitoes or not, you never know what might show up.” (P5: FGD 10: Misungwi, high malaria & high net use cluster)*

*“There are so many mosquitoes in our environment, you cannot sleep without using a mosquito net, and these mosquitoes are very tiny, but their bites are intense, often leading to skin rashes” (P6: FGD 2: Misungwi, high malaria & high net use cluster)*

Moreover, the primary reason for using LLINs was to avoid mosquito bites and malaria, but a key motivator was also the financial burden of recurrent malaria cases. LLINs are seen as a way to reduce these costs and protect family finances.

*“…I’m their sister, and I encourage them to use mosquito nets because whenever they fall sick, they come to me right away asking for money for medication.” (P9: FGD 5: Misungwi, low malaria & high net use cluster)*

### Barriers to consistent use of LLINs, acceptability, favourable, and unfavourable characteristics of LLINs

#### Adverse effects

Itching and facial burning sensations were cited as significant factors contributing to the inconsistent utilization of LLINs, particularly during the initial period of use across all LLIN types. Three months post intervention, during KAP survey, majority of users reported side effect resulting from use of nets and were more pronounced in cluster that received Interceptor (55%) and Royal Guard (48%) and less in Interceptor G2 (11%) and Olyset Plus (11%). However, there was no statistically significant difference in net usage across study arms irrespective of the side effects mentioned (p = 0.169). Contrary to qualitative findings through IDIs and FGDs that was conducted 33 months post-intervention, side effects were overwhelming attached to hindering net usage especially from Interceptor and Royal Guard arms in Misungwi.

*“One day I had a guest and I hung a mosquito net [Royal Guard]; he woke up in the middle of the night scratching his face, it felt like it was on fire. The net was itchy, he developed a skin rash, and the irritation lasted for a day before it faded.” (IDI 7, Misungwi, high malaria & high net use cluster).*

*Interviewer: What about you?*

*Participant: “…even myself, I used it when it was still new, it caused itching for three days.” (IDI 7, Misungwi, high malaria & high net use cluster).*

*“…it has too much insecticide that burns the faces and the whole body. The burning sensational can last between half an hour to three hours” (P6: FGD 11: Misungwi, high malaria & high net use cluster)*

### Bed bugs infestation

Bed bug infestations in mosquito nets emerged as a common barrier reported in both in-depth interviews and focus group discussions. Respondents commonly reported that bed bugs tend to congregate at the hanging points of the LLINs and descend during the night to feed on sleepers. Consequently, to mitigate the risk of bed bug bites, many participants opted to avoid hanging the nets altogether. The discomfort and intense itching associated with bed bug bites compelled some users to either discard their infested LLINs or subject them to washing with hot water, a practice that likely contributed to net shrinkage [30] and insecticide depletion [31]. While bed bugs are not disease vectors, their bites cause significant discomfort and distress.

*“What my colleague mentioned is true, many individuals in our community are reluctant to use mosquito nets due to the fear of bedbug infestations. For them, the discomfort of bedbug bites outweighs the risk of contracting malaria. Consequently, we are requesting that the project consider providing nets that offer protection against both mosquitoes and bedbugs. If such nets were available, no one would hesitate to use them, as it would address both concerns simultaneously, effectively eliminating the incentive to remove the nets”. (P3: FGD 3: Misungwi, low malaria & high net use cluster)*

*“…In our community, when people first began using mosquito nets, they encountered significant infestations of bedbugs. As a result, many individuals discontinued the use of the nets”. (IDI 5: Misungwi, high malaria & high net use cluster)*

However, across both the IDIs and FGDs, participants consistently avoided directly admitting to having bed bugs in their own homes, as the presence of bed bugs is culturally stigmatized and often associated with poor hygiene. Acknowledging such an infestation is seen as shameful, leading individuals to speak about the issue in a detached, third-person manner, rather than personalizing their accounts. This tendency to dissociate from the problem underscores the social stigma attached to bed bugs. Despite this reluctance to admit personal experiences, the KAP study revealed that nearly one fifth of the nets had bed bugs, with an average of 14 bed bugs per net (Table 7).

*“The belief that mosquito nets are responsible for the emergence of bedbugs has become widespread within the community, leading to numerous complaints. For instance, there was a woman who hung her mosquito nets elsewhere without using them, and when we inquired why, she responded that the nets had caused bedbugs. We took the opportunity to educate her about the environmental factors that contribute to bedbug infestations and provided her with guidance on effective measures to eliminate them. It is important to approach this issue sensitively; directly attributing bedbugs to poor hygiene can be upsetting for community members. Instead, the focus should be on educating them about practical steps to prevent bedbugs from thriving in their homes”. (KII 2: Misungwi, district level)*

### LLIN supply and perceived physical integrity

In Muleba, the pre-intervention quantitative survey revealed that barriers to consistent LLIN use included insufficient supply (56.5%) and LLINs being too torn (29.4%) (Table 5).

**Table 5:** Attitudes towards LLIN characteristics and practices in Muleba and Misungwi

Indicators	Muleba Pre-intervention: (Dec 2014)	Misungwi Post-intervention: (2020–2022)
Mosquito net colour preference	% (n)	% (n)
Dark colours(blue/green/black/red)	61.0 (339)	92.8 (2539)
Any colour	34.2 (190)	2.7 (74)
Light colours (white/yellow)	4.9 (27)	4.5 (123)
Net shape preference	% (n)	% (n)
Rectangular/square	61.4 (344)	90.7 (2482)
Any shape	22.3 (125)	2.6 (70)
Conical (round)	16.3 (91)	6.7 (184)
How do you decide that the net has failed?	% (n)	% (n)
The bed net has too many holes	71.5 (397)	88.2 (4165)
There are many mosquitoes inside the net	7.6 (42)	7.7 (361)
The bed net is dirty	6.7 (37)	4.2 (196)
The net is too olds	14.2 (79)	0 (0)
What do you do when a net has failed?	% (n)	% (n)
Discard the net	49.1 (273)	37.7 (1778)
Repair and reuse the net	31.5 (175)	27.4 (1293)
Use for alternative purposes	19.4 (108)	34.9 (1651)
What alternative purposes do you use the net for?	% (n)	% (n)
Ropes	0 (0)	54.5 (1034)
Used as bedsheet/mattress	47.7 (51)	28.6 (543)
Cover holes in the wall(s)/curtains/stuff eaves	37.4 (40)	7.4 (140)
Enclosing poultry	11.2 (12)	6.3 (120)
Collecting winged termites	14.9 (16)	0 (0)
How often do you sleep under the net?	% (n)	% (n)
Always ‘right now’	47.8 (266)	94.0 (548)
Never ‘right now’	27.1 (151)	3.6 (21)
Sometimes	25.1 (140)	2.4 (14)
Why do you sometimes or never sleep under the net?	% (n)	% (n)
Not enough nets	56.5 (144)	20.0 (5)
Net worn out/too torn	29.4 (75)	8.0 (2)
Other reasons	14.1 (36)	72% (18)

Across all IDIs and FGDs, respondents consistently cited excessive damage to the study LLINs as a primary constraint to LLIN use. In Misungwi, where both polyethylene and polyester LLINs were distributed, polyethylene LLINs (specifically Olyset Plus) were most frequently mentioned as being prone to damage.

*“I noticed that this net [Olyset Plus] tears easily, which is why it’s no longer available in many households. Even a slight squeeze on the bed can cause it to rip. (P1: FGD Misungwi, low net usage cluster)*

### Perceptions (infertility, impotence and poor sexual drive)

Cultural beliefs also played a role, with some respondents citing that LLINs could cause impotence or infertility.

*“You know, some people spread misinformation, claiming in front of others that mosquito nets negatively affect male sexual performance… (laughter)”. (IDI 3: Misungwi, high malaria village).*

*“…They believe that using mosquito nets can diminish sexual drive in men, and they also claim that women who sleep under them may struggle to conceive”. (IDI 4: Misungwi, low malaria & low net usage cluster)*

However, other participants in the same focus group discussions dismissed this belief, arguing that a lack of male sexual drive is a personal issue and unrelated to the use of LLINs, seeing it instead as an individual problem.

*“…(amidst loud group laughter)… There is no such thing; that is a personal problem. If the drive isn’t there, it simply isn’t there. If you’re unwell, that’s your individual issue. The nation will not succumb to malaria because of such a misguided belief” (P4: FGD 6: Muleba, high malaria & low net usage cluster).*

*“…that is a personal issue. I’ve been using mosquito nets for years, yet my performance [referring to sexual drive] remains strong, and my wife continues to bear children. In fact, we just welcomed a baby girl two months ago (followed by loud and hearty group laughter)” (P2: FGD 3, Misungwi, low malaria & high net use cluster).*

### Seasonality of net use

Some respondents indicated a belief that the mosquito population had declined, reducing the perceived necessity of using LLINs. This belief is consistent with the common perception that mosquitoes are absent during the dry season.

*“…For instance, I once visited my uncle and noticed that they weren’t using nets. I asked them, ‘Why aren’t you using nets?’ They responded that there are no mosquitoes anymore. I explained that this isn’t true because mosquitoes come and bite you at midnight while you’re asleep, making it difficult to notice. I then asked whether they had recently been sick with malaria, and he replied, ‘We’re puzzled about why we’re getting malaria when there are no mosquitoes.’ I told them that mosquitoes are indeed present, and therefore, they need to use nets.” (P9: FGD 5: low malaria & high net usage cluster)*

Another barrier to LLIN use mentioned in Misungwi was discussed by married women- that husbands returning late from drinking impeded use of the nets.

### Acceptability, favourable, and unfavourable characteristics of LLINs

Net acceptability, measured by the proportion of users reporting their LLINs as no longer protective, varied significantly across products at all assessed timepoints (p < 0.0001 for 24, 30, and 36 months). Olyset Plus consistently had the highest dissatisfaction levels (17.8%, 12.4% and 15.2%) followed by Royal Guard (11.7%, 7.5%, and 13.4%), while Interceptor G2 and Interceptor maintained the lowest reports (5.7–9.3%) (Additional file 3).

As shown in Table 5, blue LLINs were the most preferred colour by respondents in both districts. In Muleba the colour preference was 61% for dark colours (blue, green, black and red), 4.9% for light colours (white and yellow) and 34.2% feeling colour indifferent (Table 5). In Muleba, colour preference did not significantly differ by sex (p = 0.9982), or age group (p = 0.6921), but by education status (p = 0.0229). Those with primary or higher education had slightly more preference for light coloured nets (22% vs 16%). In Misungwi 93% of the respondents preferred dark coloured nets. This colour preference did not differ by socio-economic status (p = 0.1360).

*“I prefer the blue mosquito net because it holds up well against dust. With our red soil and uncemented floors, it’s a relief to have a net that stays clean and doesn’t show dirt easily.”. (P8: FGD 2, Misungwi, high malaria & high net use cluster).*

Rectangular LLINs were preferred by 61.4% of respondents in Muleba and 90.7% in Misungwi (Table 5). In Misungwi, where both polyester and polyethylene nets were distributed, the KAP survey participants indicated a strong preference for polyester nets 86% (2338/2736) over polyethylene alternatives 11% (86/2736).

### Comparing study nets with the nets they had previously

In both sites, participants were asked to compare the study nets with the nets they had previously. In the quantitative surveys, Muleba (Table 6) and Misungwi (Table 7), no much differences were observed across study net type. However, the qualitative finding in Misungwi revealed that there were differences when people were asked to rate study nets against the nets they owned/used in the past. Interceptor G2 consistently received positive feedback for its physical features and effectiveness in reducing malaria among children.

*“This mosquito net [Interceptor G2] does not get damaged easily, it last up to four years before showing wear. It’s soft, easy to wash, and effectively prevents mosquito penetration.” (P3: FGD 7, high malaria & high net use cluster)*.

**Table 6:** Comparing perceptions over Olyset Plus, Olyset nets and IRS in the Muleba district (data from KAP survey collected 3 months after post-intervention)

	Muleba	
How do you compare project LLINs to your previous LLINs?	Olyset net: % (n)	Olyset plus: % (n)
This LLIN is better	62.2 (166)	72.7 (216)
The LLIN is the same as previous nets	26.6 (71)	17.5 (52)
This LLIN is worse	3.8 (10)	2.7 (8)
Don’t know	7.5 (20)	7.1 (21)
Why is this project LLINs better than your previous LLINs?	Olyset net	Olyset plus
This round the nets have strong insecticide	58.1 (97)	61.8 (134)
This round the nets are larger	18.6 (31)	16.1 (35)
This round premade holes in the net are smaller	13.2 (22)	14.8 (32)
This round the nets are durable	9.6 (16)	5.5 (12)
Other	0	1.8 (3)
How long does the insecticide last on LLINs or IRS walls?	IRS	LLINs
One year or less	22.9 (136)	19.7 (117)
Two years	1.7 (10)	2.5 (15)
Three years	1.7 (10)	2.2 (13)
Four years	0.3 (2)	0.5 (3)
Five years or more	5.9 (35)	14.3 (85)
Don’t know	67.5 (400)	60.7 (360)

**Table 7:** Comparing study nets in Misungwi district (data from KAP survey)

Variables	Royal guard: % (n)	Olyset plus: % (n)	Interceptor: % (n)	Interceptor G2: % (n)
How do you compare project nets to your previously used nets?				
This net is better	59.4 (590)	48.4 (366)	69.3 (802)	66.1 (869)
They are similar	28.4 (282)	38.4 (291)	23.6 (273)	28.7 (377)
This net is worse	6.8 (68)	7.5 (57)	2.2 (25)	1.5 (20)
Don’t know	5.4 (54)	5.7 (43)	4.9 (57)	3.7 (49)
Why is this net better?				
Mosquitoes cannot penetrate	80.5 (494)	74.8 (294)	71.7 (616)	69.2 (659)
The net is soft	7.3 (45)	9.2 (36)	12.0 (103)	10.2 (97)
Does not acquire holes easily	5.2 (32)	4.8 (19)	4.9 (42)	8.2 (78)
It is new	1.1 (7)	0.8 (3)	0 (0)	0 (0)
Does not itch	0.2 (1)	0.3 (1)	0.6 (5)	0.2 (2)
Children don’t get malaria	1.0 (6)	1.8 (7)	0.1 (1)	0.4 (4)
Don’t know/other	4.7 (29)	8.4 (33)	9.5 (82)	11.8 (112)
Proportion of nets observed with bed bugs: % (n/N)	19.4 (87/448)	19.0 (58/305)	28.7 (218/761)	22.2 (157/707)
Mean number of bed bugs per LLIN observed by field worker [95% CI]^a^	14.2 [12.1–16.3]	14.3 [11.7–16.9]	14.8 [13.4–16.1]	15.4 [13.7–16.9]

^a^We capped bed bug counts at 30

Olyset Plus was distributed across both districts, with user perceptions differing by location. Olyset Plus was slightly preferred to Olyset net in Muleba (62.2% vs 72.2%), p = 0.1395, due to its favourable qualities (strong insecticidal properties) (Table 6). In Misungwi, in the qualitative study, participants expressed concerns about its physical integrity, noting that Olyset Plus did not physically last as long as expected.

*“This net [Olyset plus], is good, but it tears easily.” (P7: FGD Misungwi, low net use cluster)*

In Misungwi, both Royal Guard and Interceptor nets were noted to cause adverse effects like skin irritation and facial burning. These negative experiences led to a decrease in LLIN use among some respondents especially in the immediate days after distribution.

*“Due to the negative impact of the net [in Royal Guard arm], such as causing rashes when first used, people stopped using them entirely” (P11: FGD 7, high malaria & high net use cluster)*.

### Repurposing of LLINs

**Fig. 2:**
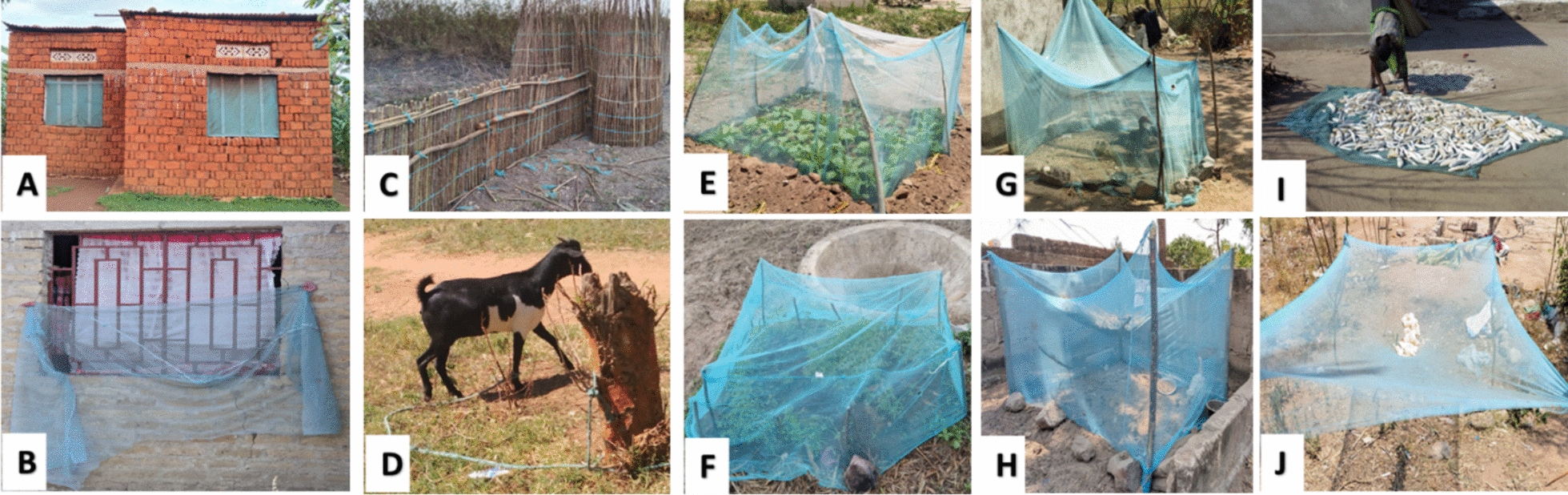
Repurposing of LLINs: Beneficial repurposing (window screening) (**A**–**B**), torn into strips for making fishing traps and ropes (**C–D**), protecting seedling and young plants (**E**–**F**), for making poultry coops (**G–H**), drying cassava (**I**–**J**)

In both Muleba and Misungwi, participants reported repurposing LLINs for various tasks, including making ropes, protecting crops, and screening windows (Fig. 2). For example, 54.5% of respondents in Misungwi used LLINs as ropes, and 28.6% used them as bed sheets or mattresses (Table 5).

*Mosquito nets are used to protect seedlings (P3), poultry coops (P4) and making ropes because they do not cut easily (P7) (FGD 2: Misungwi, high malaria, and high net use cluster)*

It was also observed that local government leaders at the ward and village levels have implemented strict measures to control malaria. These measures include preventing the misuse of nets and ensuring that households maintain cleanliness around their surroundings to eliminate potential mosquito breeding sites.

*“I was fined for tying six bags of scarlet eggplant ‘nyanya chungu’ with net strings at Buhongwa market [a famous farmers market]” (P7: FGD 7, high malaria & high net use cluster).*

*Interviewer: Who fined you, and how much did you pay?*

*“An environmental health officer came by and questioned who permitted me to use a net for tying my bags. I ended up having to pay two thousand Tanzanian shillings per bag as a fine, so I’ve decided never to use those nets again, except for sleeping under them” (P7: FGD 7, Misungwi, high malaria & high net use cluster)*

*“Last month, we prosecuted some individuals who refused to participate in community cleaning activities and failed to cover ponds around their homes, these actions are crucial for preventing mosquito breeding and controlling malaria” (KII 5, Muleba, ward level).*

### Lead researcher (Eliud Lukole’s 6.14 kms observational walk)

**Fig. 3:**
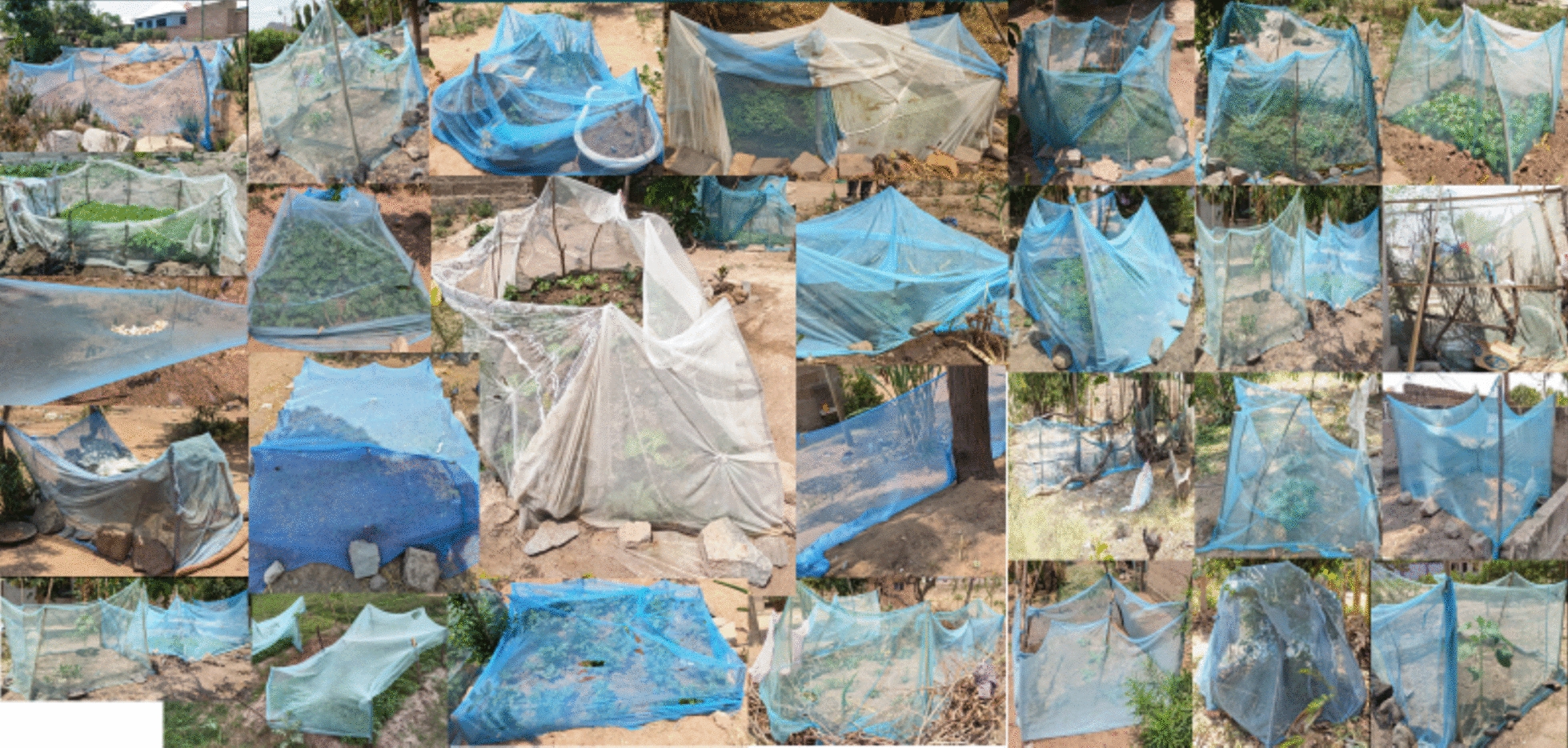
Images of re-purposed nets from Eliud Lukole’s 6.14 km walk

**Fig. 4:**
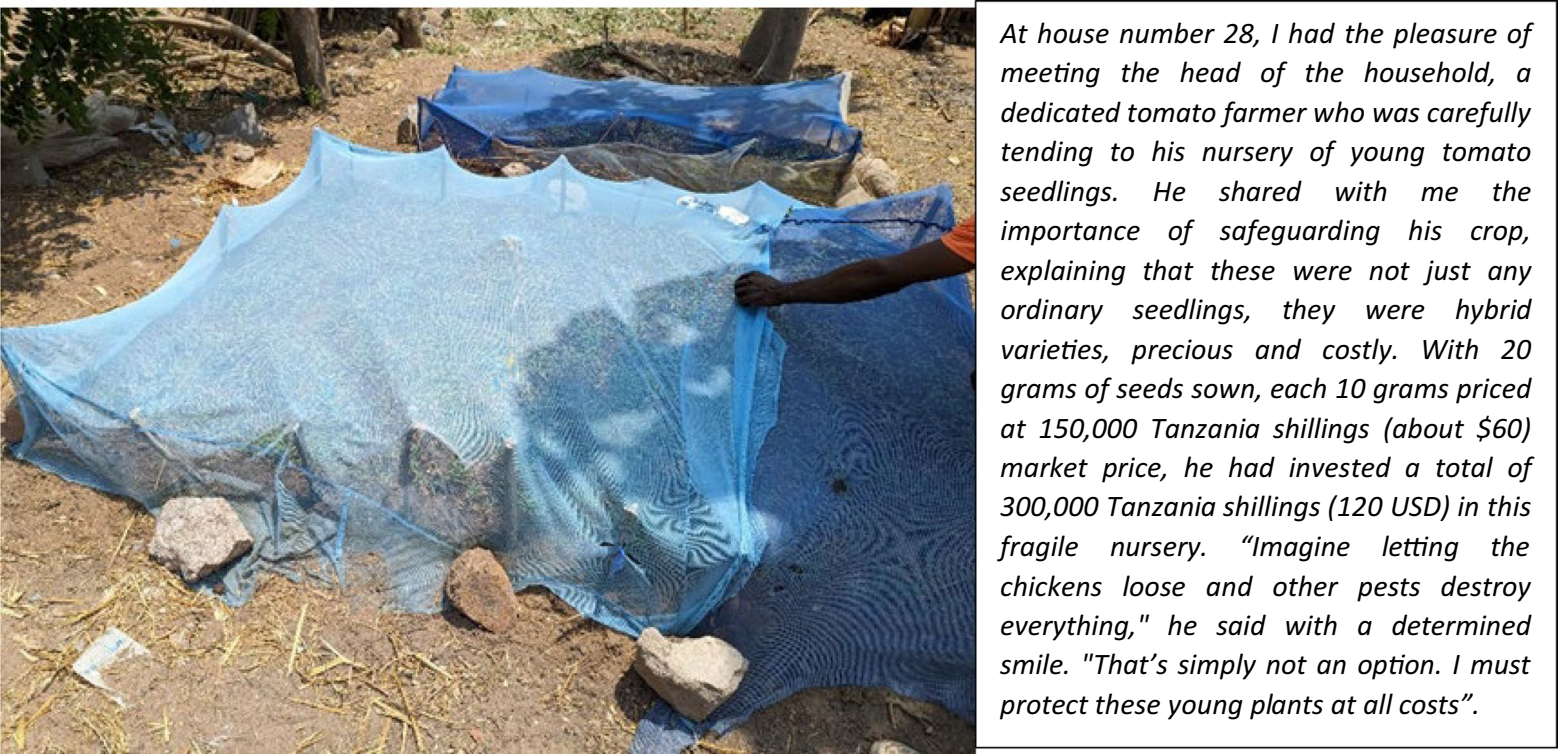
Tomato nursery at house number 28

In November 2021, same time when the qualitative study was conducted in the Misungwi district, the lead researcher (EL) took the opportunity to explore various alternative uses of nets in 43 households in one of the hamlets. As the researcher walked through the area (Additional file 5), he used a handheld GPS to record geolocations and a phone to take photographs of repurposed LLINs, especially those where whole or large sections were utilized. The researcher engaged with residents, asking about the reasons behind each specific use. The majority of these LLINs were employed to cover crops or as barriers around areas where crops were planted or as chicken coops (Fig. 3). At household number 28, the researcher had an extended conversation with a potato farmer who was tending to his tomato nursery (Fig. 4). The collected images were then superimposed onto a map, corresponding to their geolocations, to illustrate the proximity of these households (Additional file 5).

### Increasing LLIN use: participant recommendations

Participants provided key recommendations for maintaining LLIN use. Regular awareness programmes should be tailored to local languages to reach both literate and illiterate populations, addressing misconceptions and educating on malaria, LLIN benefits, and proper use. Training and incentives for community health workers (CHW) to conduct door-to-door education were also recommended to boost programme coverage, while village meetings were discouraged due to low attendance.

*“They [CHWs] are volunteers, but their work is challenging, and they need some motivation. Please support them during malaria activities, as they often work in harsh environments. For instance, during a COVID-19 pandemic, the DMO [District medical officer] offered ten thousand shillings to CHWs for vaccinating ten people”. (KII 2, Misungwi, ward level)*

### Distinct differences between Misungwi and Muleba that might influence potential repurposing of LLINs

Both districts had similar exposure to SBCC messages and comparable knowledge of malaria prevention (Additional file 2). However, Misungwi households had more LLINs at baseline (62.3% access of 1 LLIN per sleeping space used last night, and 61% usage) than Muleba (16.9% access and 27.1% usage) (Additional file 7), and cultural, lifestyle, and SES differences between the districts may have influenced LLIN use and repurposing (Additional file 6).

## Discussion

This study evaluated community perceptions of malaria prevention, and the acceptability and preference of dual-AI LLINs compared to standard LLINs (Olyset net and Interceptor) in Muleba and Misungwi districts, Tanzania, where these novel nets were introduced. Acceptability, defined as the extent to which nets are perceived as suitable and effective by users, was high for dual-AI LLINs but varied across net types. Interceptor G2 was the most preferred net in Misungwi due to its perceived physical integrity, soft texture, and superior effectiveness in reducing malaria, particularly among children. By contrast, Olyset Plus, though perceived as effective, was less favoured in Misungwi due to frequent tearing. In Muleba, where only polyethylene nets have been distributed, Olyset Plus was well received for its perceived strong insecticidal properties compared to the standard Olyset net. Side effects were associated with certain brands of nets, with participants reporting skin irritation, burning, and itching linked to Royal Guard and Interceptor. Net preferences were primarily driven by durability, comfort, and efficacy, underscoring the need for user centred designs to optimize acceptability and consistent usage.

Participants frequently reported side effects associated with Royal Guard (48%) and Interceptor (55%), likely due to higher concentrations of alpha-cypermethrin, as reported in other studies [32–34], which is known to cause more side effects than permethrin [33, 35, 36]. A Benin RCTs [37] also reported similar transient adverse events, such as itching and mild skin irritation, associated with alpha-cypermethrin with 63.8% of Interceptor users and 52.5% of Royal Guard users, reporting side effects [27, 37]. These numbers were higher than those reported in Liberia [38], where only 17.5% reported issues with Interceptor LLINs. The quantitative findings in this study showed that while side effects were commonly cited, they did not deter LLIN usage in most households. This may be because side effects are short-lived and typically resolve within a few days of use, as well as the protective benefits of the nets against malaria, which participants emphasized, outweighing any discomfort. However, in a small subset of users, these effects did lead to reduced adherence or even non-use. Given these findings, adverse events associated with LLIN use during distribution campaigns should be monitored. Clear communication about the transient nature of most side effects and strategies to mitigate discomfort (e.g., washing nets before use, as recommended to participants in this study) may further enhance user compliance and satisfaction.

The dissatisfaction reported in among users of Olyset Plus reporting their nets ineffective could be paralleled with the decline in their durability observed in bio efficacy [31], physical integrity [30, 31]. The decline in perceived protection may have contributed to reduced net use over time which was more pronounced in Olyset Plus cluster [30, 39], with only 16% of study nets still in use at 36 months and just 19% of households maintaining sufficient net coverage. This convergence of biological degradation, physical wear, and declining user confidence highlights the critical role of both technical durability and behavioural adherence in sustaining LLIN effectiveness. Products that combine chemical longevity with user acceptability driven by perceived comfort, net shape, colour, and continued protection are more likely to retain high usage over time and achieve durable epidemiological impact.

In this study, LLINs were primarily used when mosquito-biting nuisance was high or malaria was perceived as a significant threat. In villages with high biting nuisance, participants continued to use LLINs even if they do not like certain features such as the fabrics, whereas in low biting nuisance areas these characteristics resulted in inconsistent or non-use. Those findings align with evidence from Solomon Islands where participants in areas with high and perennial mosquito nuisance reported consistent LLIN use despite unfavourable net attributes, whereas those in low or seasonal transmission settings more often cited discomforts as justification for intermittent or non-use [9]. Previous studies have similarly linked high mosquito densities and perceived malaria risk with LLIN compliance [40, 41]. Educational programmes should emphasize that the risk of malaria persists even when mosquito numbers are low and should be consistently use for better impact [42].

Repurposing of LLINs was notably more prevalent in Misungwi compared to Muleba, and this pattern appears closely tied to the baseline net coverage and subsequent net distribution dynamics. At baseline, households in Misungwi had significantly higher LLIN access (65.8% population access) and usage (61%)[27] than those in Muleba (37% access and 27.5% usage) [26]. More so, Misungwi received PBO LLINs earlier through the School Net Programme (SNP), prior to the main study mass campaign, which may have contributed to net saturation and the perception that new nets would continue to be distributed frequently. This oversupply appears to have created reduced perceived value of LLINs, fostering a disposable attitude and leading to increased misused nets being repurposed for non-malaria-related uses [43]. This is further supported by qualitative findings showing that households in Misungwi were more likely to treat nets as replaceable goods due to their abundance and expectation of continued supply. Moreover, this behavioural trend is reflected in net survivorship data: although 92% of households in Misungwi owned at least one study net after 12 months, ownership dropped to 62% by month 36, and actual study net usage declined from 72 to 23% over the same period [39]. In contrast, net survival and use in Muleba where baseline access and use were lower, was relatively better maintained. This suggests that excessive initial coverage, without reinforcing value through social behaviour change communication (SBCC), may paradoxically erode long-term net survival and use.

While knowledge about malaria prevention was high among participants, with over 90% demonstrating a clear understanding of key concepts, the practical application of this knowledge often varied. This highlights the gap between knowledge and behaviour, emphasizing the role of contextual and cultural factors in LLIN use. Evidence suggests that knowledge alone is rarely sufficient to drive or sustain behavioural change [44]; contextual factors such as environmental conditions, cultural norms, and competing priorities significantly influence compliance. For example, despite high levels of knowledge, some participants reported reduced LLIN use during the hot season, citing discomfort as a barrier.

Preferences for LLINs vary greatly across settings, with a strong preference for blue, rectangular LLINs observed in this study, aligning with similar findings from Kenya [23, 24]. The widespread preference for blue nets stems from prolonged distribution via national distribution programmes, which have fostered familiarity and acceptance. Moreover, in dwellings with mud walls and uncemented floors, blue nets are perceived to conceal dirt more effectively, thereby minimizing the frequency of washing. Rectangular nets are considered more practical by many households as they are easier to tuck under mattresses, offer broader surface coverage, and better accommodate multiple sleepers a common scenario in high-density sleeping arrangements. In contrast, regions such as Ethiopia [45, 46] and Senegal [47] exhibit a marked preference for conical LLINs, primarily attributed to their ease of installation and efficient space utilization. This preference underscores the influence of cultural practices, housing structures, and user experience on LLIN adoption, highlighting the need for context-specific design considerations in vector control interventions.

The study identified prevalent misconceptions about malaria transmission, including beliefs linking it to contaminated water, bad air, sun exposure, or superstition. Some participants also perceived LLINs as attracting bed bugs similar to findings in Kenya [23], a concern that warrants targeted education [48, 49]. While LLINs are not designed to target bed bugs, pyrethroid-treated nets have shown some suppressive effects [50]. However, multiple studies have reported varying degrees of pyrethroid resistance in bed bug populations [51–55]. The comparable infestation rates observed across study arms likely reflect the limited efficacy of current LLINs under prevailing bed bug pressure, a situation that warrants targeted entomological investigation to better characterize resistance patterns and inform control strategies.

This study has several limitations. First, its qualitative nature limits the representativeness of findings for the wider population. Nonetheless, the inclusion of quantitative data from two districts helps provide a broader perspective. Second, recall bias regarding previously used nets may have influenced participants’ comparisons. This effect was largely mitigated by the balanced design of the RCTs, which distributed such biases evenly across study arms. Third, the observed variation in the desirability of Olyset Plus between Muleba and Misungwi underscores the need for larger-scale studies. Finally, potential biases in self-reported data on LLIN ownership and usage frequency are acknowledged; however, nets were physically observed and inspected before being recorded.

## Conclusion

This study reveals that the acceptability and consistent use of dual-AI LLINs are primarily driven by perceived efficacy, fabric quality, mosquito nuisance, and comfort. Issues like physical integrity, bed bugs, and adverse reactions hinder optimal use. Generating entomological data on non-target pests such as bed bugs could inform net selection and improve uptake. Educational programmes focused on adult men, the primary non-users, could improve net utilization. Since education level shapes colour preferences and malaria misconceptions, integrated campaigns are needed to dispel myths and promote consistent use. Incorporating user perception metrics into LLIN evaluation and procurement strategies will further align interventions with community needs.

## Supplementary Information

Additional file 1.

## Data Availability

The datasets generated and/or analysed during the current study are not publicly available due strict laws in Tanzania that restrict data to be shared outside the country without a Data Transfer agreement (DTA.pdf (nimr.or.tz)). For the interested researchers, the DTA can be completed by the help of Dr. Eliud Lukole (ellyluf@ymail.com) or Prof. Natacha Protopopof (natacha.protopopof@swisstph.ch).
