# Supplementary material for: Acceptability and preferences for dual-active ingredient long-lasting insecticidal nets in rural Tanzania: a mixed-methods study
Source: Malar J. 2025 Dec 15;25:44. doi: 10.1186/s12936-025-05723-w (PMC12822049; doi:10.1186/s12936-025-05723-w)
Supplement: Supplementary file 1 — Additional file 1. [file 12936_2025_5723_MOESM1_ESM.docx]

**Supplemental information**

Additional file 1: Distribution of villages where focus groups and in-depth interviews were conducted: by levels of LLIN usage, and malaria prevalence per study arm

| **Region** | **District** | **Study arm** | **Net usage levels** | **Villages/hamlets** | **Malaria prevalence** | **Villages/hamlets** |
| --- | --- | --- | --- | --- | --- | --- |
| Mwanza | Misungwi | Olyset Plus | Low (≤40%) | Budutu, Mwajombo, Lukanga, Nyambiti | Low (≤30%) | Mwaholo |
|  |  |  | High (>40) | Ibongoya A, Ibongoya B, Ngwa’mazengo, Lubili A | High (>30) | Ibongoya A, Ibongoya B, Lubili A |
|  |  | Royal Guard | Low (≤40%) | Nya’nghomango, Lubili B, Busongo | Low (≤30%) | Masawe, Busolwa |
|  |  |  | High (>40) | Mwagala, Mamaye, Masawe, Gulumungu, | High (>30) | Mwagala, Lubili B, Gulumungu, Busongo |
|  |  | Interceptor G2 | Low (≤40%) | Maganzo, Mbalama, Kifune | Low (≤30%) | Mbela |
|  |  |  | High (>40) | Magaka, Mbela, Ngwa’mbola, Ngwa’mazengo | High (>30) | Mbalama, Ngwa’mazengo, Kifune |
|  |  | Interceptor | Low (≤40%) | Gambajiga, Ngh’amve | Low (≤30%) | Nghamve |
|  |  |  | High (>40) | Igongwa , Kwimwa | High (>30) | Gambajiga, Igongwa , Kwimwa, Ilalambogo |
| Kagera | Muleba | Olyset Net | Low (≤40%) | Kakoma, Nyakatanga | Low (≤30%) | Kyanshenge |
|  |  |  | High (>40) | Kabirizi, Kyanshenge | High (>30) | Kakoma, Kabirizi,  Nyakatanga |
|  |  | Olyset Plus | Low (≤40%) | Ngenge, Biija | Low (≤30%) | Biija |
|  |  |  | High (>40) | Maigibili | High (>30) | Maigibili, Ngenge, |
|  |  | Olyset Net + IRS | Low (≤30%) | Rulanda | Low (≤30%) | Rulanda |
|  |  |  | High (>40) | Kamatoju | High (>30) | Kamatoju |
|  |  | Olyset Plus + IRS | Low (≤40%) | Kashanda | Low (≤30%) | Kashanda |
|  |  |  | High (>40) | Kyamyorwa, Kangoma, | High (>30) | Kyamyorwa, Kangoma, |

Additional file 2: Knowledge of malaria and preventive practices in Muleba and Misungwi (data from KAP surveys)

|  | Muleba | |  | Misungwi |
| --- | --- | --- | --- | --- |
|  | **Baseline (Dec 2014): % (n=560)** | **Post (April 2015): % (n=593)** |  | **Post-intervention (2020-2022)** |
| How is malaria transmitted to humans?** | | | | |
| Bite of an infected mosquito | 92.5 (518) | 91.7 (544) |  | 86.6 (2,106) |
| Others | 27.9 (156) | 38.9 (230) |  | 15.7 (382) |
| Ways to prevent malaria** | | | | |
| Sleeping under LLINs | 90.5 (507) | 90.6 (537) |  | N/A |
| Spraying insecticide inside the house | 30.7 (172) | 43.5 (258) |  | N/A |
| Others | 43.2 (242) | 35.5 (210) |  | N/A |
| Personal protection measures against malaria | | | | |
| Mosquito nets | 87.7 (490) | N/A |  | N/A |
| Closing windows and doors | 15.9 (89) | N/A |  | N/A |
| Gauze wire in windows | 7.9 (44) | N/A |  | N/A |
| Mosquito coil | 4.3(24) | N/A |  | N/A |
| Where do you first seek malaria treatment? | | | | |
| Hospital, health center, or dispensary | 57.1 (320) | 76.1 (451) |  | 45.4 (1,535) |
| Drug shop/pharmacy/general shop | 35.9 (201) | 18.7 (111) |  | 54.0 (1,823) |
| Traditional healer (herbalist) | 5.5 (31) | 3.9 (23) |  | 0.3 (9) |
| Community Health Worker | 0.5 (3) | 0.3 (2) |  | 0.21 (7) |
| Traditional healer (spiritual) | 0.4 (2) | 0 |  | 0 (0) |

| How often do you sleep under the LLIN? | | | | |
| --- | --- | --- | --- | --- |
| Always ‘right now’ | 47.8 (266) | 94.0 (549) |  | N/A |
| Never ‘right now’ | 27.2 (151) | 3.6 (21) |  | N/A |
| Sometimes | 25.0 (140) | 2.4 (14) |  | N/A |
| Why do you sometimes or never sleep under the LLIN? | | | | |
| Not enough nets | 144 (56.5%) | 5 (20%) |  | N/A |
| Net worn out/too torn | 75 (29.4%) | 2 (8%) |  | N/A |
| Other reasons | 36 (14.1%) | 18 (72%) |  | N/A |

Additional file 3: Proportion of participants reporting nets as no longer protective

| **Variables** | **24 months: % (n/N)** | **30 months: % (n/N)** | **36 months: % (n/N)** |
| --- | --- | --- | --- |
| Other nets | 11.3 (412/3654) | 6.7 (198/2972) | 6.5 (273/4204) |
| Interceptor G2 | 6.8 (90/1315) | 5.7 (47/832) | 8.4 (58/690) |
| Olyset Plus | 17.8 (135/757) | 12.4 (51/411) | 15.2 (44/289) |
| Royal Guard | 11.7 (116/994) | 11.0 (67/610) | 13.4 (57/427) |
| Interceptor | 6.0 (69/1157) | 7.5 (59/790) | 9.3 (65/710) |

Additional file 4: Proportion of participants reporting side effects caused by the LLINs

| **Variables** | **Interceptor G2: N=162** | **Olyset Plus: N=162** | **Royal Guard: N=168** | **Interceptor: N=164** |
| --- | --- | --- | --- | --- |
| Participants with side effects: % (n) | 10.5 (17) | 10.5 (17) | 47.6 (80) | 54.9 (90) |
| Type of side effect experienced: % (n) |  |  |  |  |
| Skin irritation or paraesthesia | 70.6 (12) | 52.9 (9) | 63.8 (51) | 67.8 (61) |
| Facial burning | 5.9 (1) | 41.2 (7) | 31.3 (25) | 27.8 (25) |
| Feeling sick or nausea | 0 (0) | 0 (0) | 0 (0) | 1.1 (1) |
| Headache | 11.8 (2) | 0 (0) | 0 (0) | 0 (0) |
| Other | 11.8 (2) | 0 (0) | 0 (0) | 1.1 (1) |
| Runny eyes or nose or sneezing | 0 (0) | 5.9 (1) | 5.0 (4) | 2.2 (2) |

*Additional file 5: Eliud Lukole's 6.14 km GPS-track and superimposed pictures*


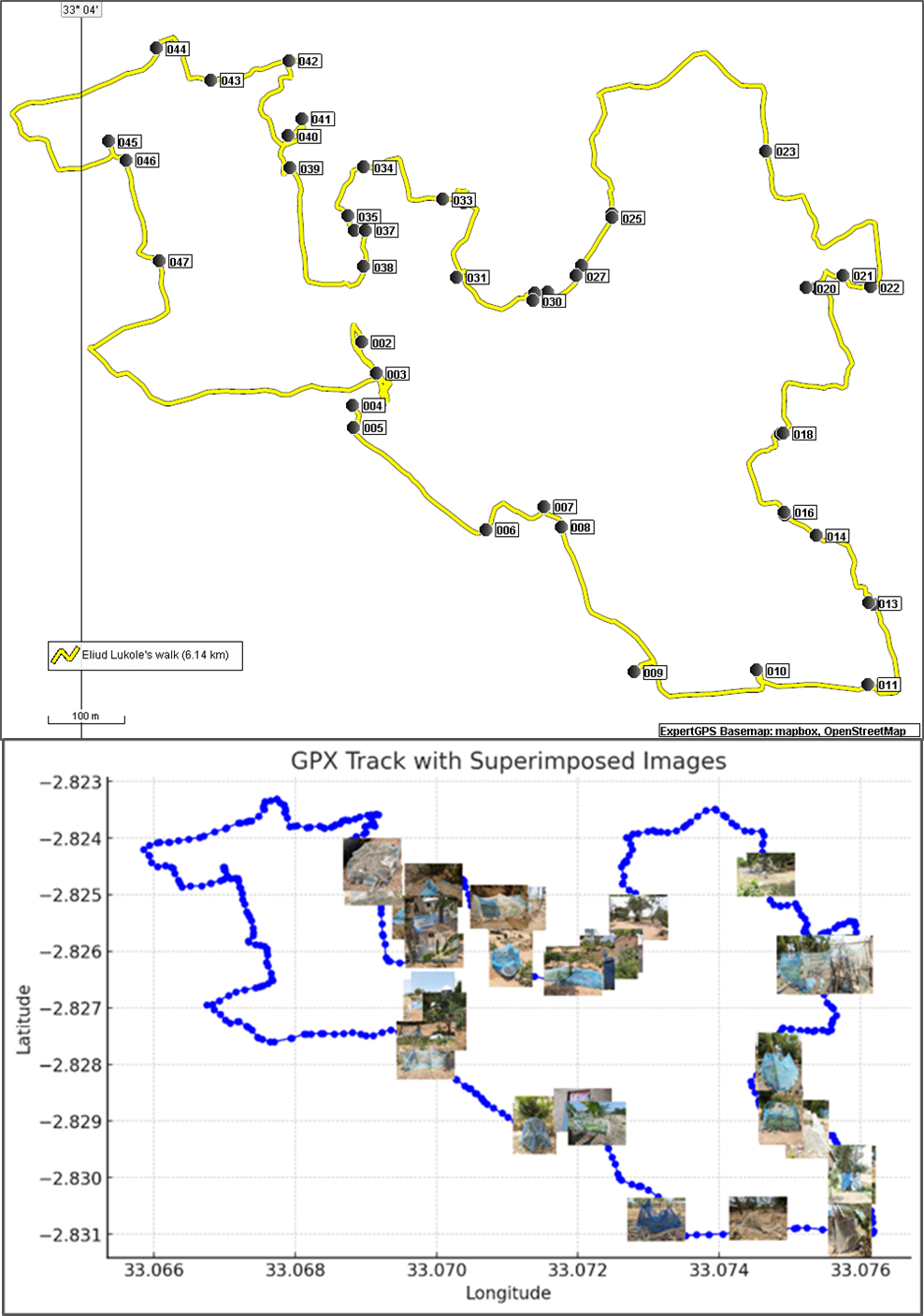


*Additional file 6: Differences between Muleba and Misungwi districts*

| **Category** | **Muleba** | **Misungwi** |
| --- | --- | --- |
| Ethnic Composition | Predominantly Haya, with a rich history in coffee and banana farming, and traditional iron smelting. | Predominantly Sukuma, strong agricultural and pastoral culture, focus on cotton and cattle herding. |
| Crop Types | Annual, less frequent farming needed | Perennial crops like rice, sweet potatoes, cassava, and sorghum, require seasonal farming and vegetation clearance. |
| Climate & Landscape | Greener, cooler, wetter, tropical rainforest climate, hilly, forested, nucleated settlements. | Hotter, drier, tropical savanna climate, flat landscape, scattered settlements, sparse tree cover. |
| Soil Fertility | More fertile soils, lush vegetation, farming done by hand hoe. | Less fertile soils, farming with ploughs drawn by cattle. |
| Housing | Traditional houses are made of bricks/mud and poles, thatched with banana leaves or dry barks, and tin roofs. | Simple houses made of mud bricks, thatched roofs, and increasing use of iron sheets. |
| Resource Use | Banana dry barks are used as ropes, less need for synthetic ropes. | With limited plant resources, LLINs were repurposed as ropes for various uses, including tying cattle yokes. |
| Crop Types | Annual, less frequent farming is needed. | Perennial crops like rice, cotton, and sorghum, require seasonal farming and vegetation clearance. |
| Literacy Rate | Relatively higher, better access to education due to historical missionary activities. | Lower literacy rate, challenges in education access due to economic constraints, and dispersed population. |
| Economic Development | Moderately developed, income from agriculture and fishing, higher per capita income from coffee. | Lower socio-economic status, income from agriculture and livestock, reliance on subsistence farming. |

*Additional file 7: All net (any net) and study nets use, malaria prevalence, population access and use:access ratio in the study sites from RCTs per survey timepoint*

| **District** | **Survey timepoint** | **Transmission season** | **Mean malaria prevalence** | **Any net** | | | **Study net** | | |
| --- | --- | --- | --- | --- | --- | --- | --- | --- | --- |
|  |  |  |  | **Net use: %** | **Population access: %** | **Use:access ratio** | **Net use: %** | **Population access: %** | **Use:access ratio** |
| Muleba | Baseline | High | 65 | 27.5 | 37 | 0.74 | n/a | n/a | n/a |
| Muleba | 4 months | High | 40 | 76 | 77.45 | 0.98 | 70.5 | 75.09 | 0.94 |
| Muleba | 9 months | Low | 32 | 76 | 70.44 | 1.08 | 70.5 | 67.98 | 1.04 |
| Muleba | 16 months | High | 38 | 49 | 55.41 | 0.88 | 44 | 53.87 | 0.82 |
| Muleba | 22 months | Low | 49 | 53 | 57.62 | 0.92 | 41 | 45.88 | 0.89 |
| Muleba | 28 months | High | 72 | 59 | 53.58 | 1.10 | 41 | 42.5 | 0.96 |
| Muleba | 33 months | Low | 50 | 48 | 58.06 | 0.83 | 21.5 | 35.39 | 0.61 |
|  |  |  |  |  |  |  |  |  |  |
| **District** | **Survey timepoint** | **Transmission season** | **Malaria prevalence** | **Any net** | | | **Study net** | | |
|  |  |  |  | **Net use** | **Population access** | **Use:access ratio** | **Net use** | **Population access** | **Use:access ratio** |
| Misungwi | Baseline | High | 44.2 | 61.0 | 65.8 | 0.93 | n/a | n/a | n/a |
| Misungwi | 3 months | High | n/a | 81.8 | 93.8 | 0.87 | 72.1 | 76.5 | 0.94 |
| Misungwi | 12 months | Low | 18.7 | 83.0 | 84.3 | 0.98 | 61.8 | 55.8 | 1.11 |
| Misungwi | 18 months | High | 46.7 | 72.2 | 80.8 | 0.89 | 47.8 | 41.2 | 1.16 |
| Misungwi | 24 months | Low | 38.4 | 72.2 | 75.2 | 0.96 | 40.9 | 30.8 | 1.33 |
| Misungwi | 30 months | High | 45.6 | 62.6 | 75.5 | 0.83 | 29.6 | 30.8 | 0.96 |
| Misungwi | 36 months | Low | 31.1 | 66.7 | 76.1 | 0.88 | 20.8 | 15.4 | 1.35 |
